# Changes in the equine fecal microbiota associated with the use of systemic antimicrobial drugs

**DOI:** 10.1186/s12917-015-0335-7

**Published:** 2015-02-03

**Authors:** Marcio C Costa, Henry R Stämpfli, Luis G Arroyo, Emma Allen-Vercoe, Roberta G Gomes, J Scott Weese

**Affiliations:** Department of Pathobiology, Ontario Veterinary College, University of Guelph, Guelph, Canada; Department of Clinical Studies, Ontario Veterinary College, University of Guelph, Guelph, Canada; Department of Molecular and Cellular Biology, College of Biological Sciences, University of Guelph, Guelph, Canada; Department of Clinical Studies, “Universidade Estadual de Londrina”, Londrina, Brazil

**Keywords:** Horses, Antibiotics, Intestinal microbiota, Intestinal bacteria, Microbiome, Antimicrobial associated diarrhea

## Abstract

**Background:**

The intestinal tract is a rich and complex environment and its microbiota has been shown to have an important role in health and disease in the host. Several factors can cause disruption of the normal intestinal microbiota, including antimicrobial therapy, which is an important cause of diarrhea in horses. This study aimed to characterize changes in the fecal bacterial populations of healthy horses associated with the administration of frequently used antimicrobial drugs.

**Results:**

Twenty-four adult mares were assigned to receive procaine penicillin intramuscularly (IM), ceftiofur sodium IM, trimethoprim sulfadiazine (TMS) orally or to a control group. Treatment was given for 5 consecutive days and fecal samples were collected before drug administration (Day 1), at the end of treatment (Days 5), and on Days 14 and 30 of the trial. High throughput sequencing of the V4 region of the 16S rRNA gene was performed using an Illumina MiSeq sequencer. Significant changes of population structure and community membership were observed after the use of all drugs. TMS caused the most marked changes on fecal microbiota even at higher taxonomic levels including a significant decrease of richness and diversity. Those changes were mainly due to a drastic decrease of Verrucomicrobia, specifically the “5 genus *incertae sedis*”. Changes in structure and membership caused by antimicrobial administration were specific for each drug and may be predictable. Twenty-five days after the end of treatment, bacterial profiles were more similar to pre-treatment patterns indicating a recovery from changes caused by antimicrobial administration, but differences were still evident, especially regarding community membership.

**Conclusions:**

The use of systemic antimicrobials leads to changes in the intestinal microbiota, with different and specific responses to different antimicrobials. All antimicrobials tested here had some impact on the microbiota, but TMS significantly reduced bacterial species richness and diversity and had the greatest apparent impact on population structure, specifically targeting members of the Verrucomicrobia phylum.

**Electronic supplementary material:**

The online version of this article (doi:10.1186/s12917-015-0335-7) contains supplementary material, which is available to authorized users.

## Background

The intestinal microbiota performs important roles in the maintenance of health and on the pathophysiology of several diseases [1]. In the horse, the intestinal bacterial microbiota is particularly important due to its role in cellulose fermentation and short chain fatty acid production, which comprise the main energy sources for this animal species [2]. Gastrointestinal disease is one of the leading causes of morbidity and mortality in the horse [3], yet, despite its importance, the equine intestinal microbiota has not been extensively investigated. However, new molecular technologies, especially next-generation sequencing methods, have become more available of late, and recently a number of publications have brought new insights into this complex microbial community [4-9]. Yet, much about the equine intestinal microbiota remains to be discerned.

Several factors have been shown to induce profound changes on the gastro-intestinal microbiota of horses including diet [10,11], intestinal disease [5], fasting [12,13] and transportation [14]. Of special interest are the effects of antimicrobials, as this group of drugs can have major impact on the intestinal microbiota of horses [15], and colitis is an important (and potentially life-threatening) complication of antimicrobial exposure in this species [16-18].

Changes in the intestinal microbiota induced by the use of antibiotics can be present as soon as 24 hours after administration of the drug in humans, with profound changes around 4 days [19,20] and partial recovery of the intestinal microbiota occurring around 30 to 40 days after treatment [19,21,22]. However, structural changes in bacterial communities may take years to return to pre-treatment baseline following antibiotic induced disturbance [23].

To date, many investigations of the effects of antimicrobial usage in horses have been limited to culture-based studies [15,24,25], which have yielded conflicting results. Gustafsson et al. [25] found no effect on the fecal microbiota of horses treated with oral or intravenous trimethoprim-sulfadiazine (TMS). Conversely, Harlow et al. [15] showed dramatic disruption of the culturable microbiota concurrent with increased shedding of enteropathogens after administration of TMS or ceftiofur sodium in horses. Moreover, a study using DGGE failed to detect changes caused by the use of antibiotics [26]; however, it is unclear whether there was no true difference or whether results simply reflect the limited resolution of this technique. While culture-dependent methods are necessary to characterize new bacterial species and can give better resolution for the identification of microorganisms, sequencing methods have become the elective choice for a broader characterization of the intestinal microbiota.

The objective of this study was to evaluate changes in the intestinal microbiota of healthy horses in response to administration of commonly used antimicrobials using next generation sequencing.

## Results

### Metrics

A total of 4,275,413 reads from 96 samples (mean: 47,431; SD: 29,796) passed all quality filters and were assigned into operational taxonomic units (OTUs). A subsample of 10.482 reads per sample was taken in order to normalize the number of reads across all samples and six samples were excluded from analysis because of low read numbers. These belonged to the groups: penicillin (Day 30), TMS (Day 1: two samples and Day 14: two samples) and control (Day 5). The number of OTUs in the subsampled population varied between 1,333 and 3,628 per sample (mean: 2,671; SD: 488). The number of OTUs found in each sample is presented in Additional file 1.

**Figure 1:**
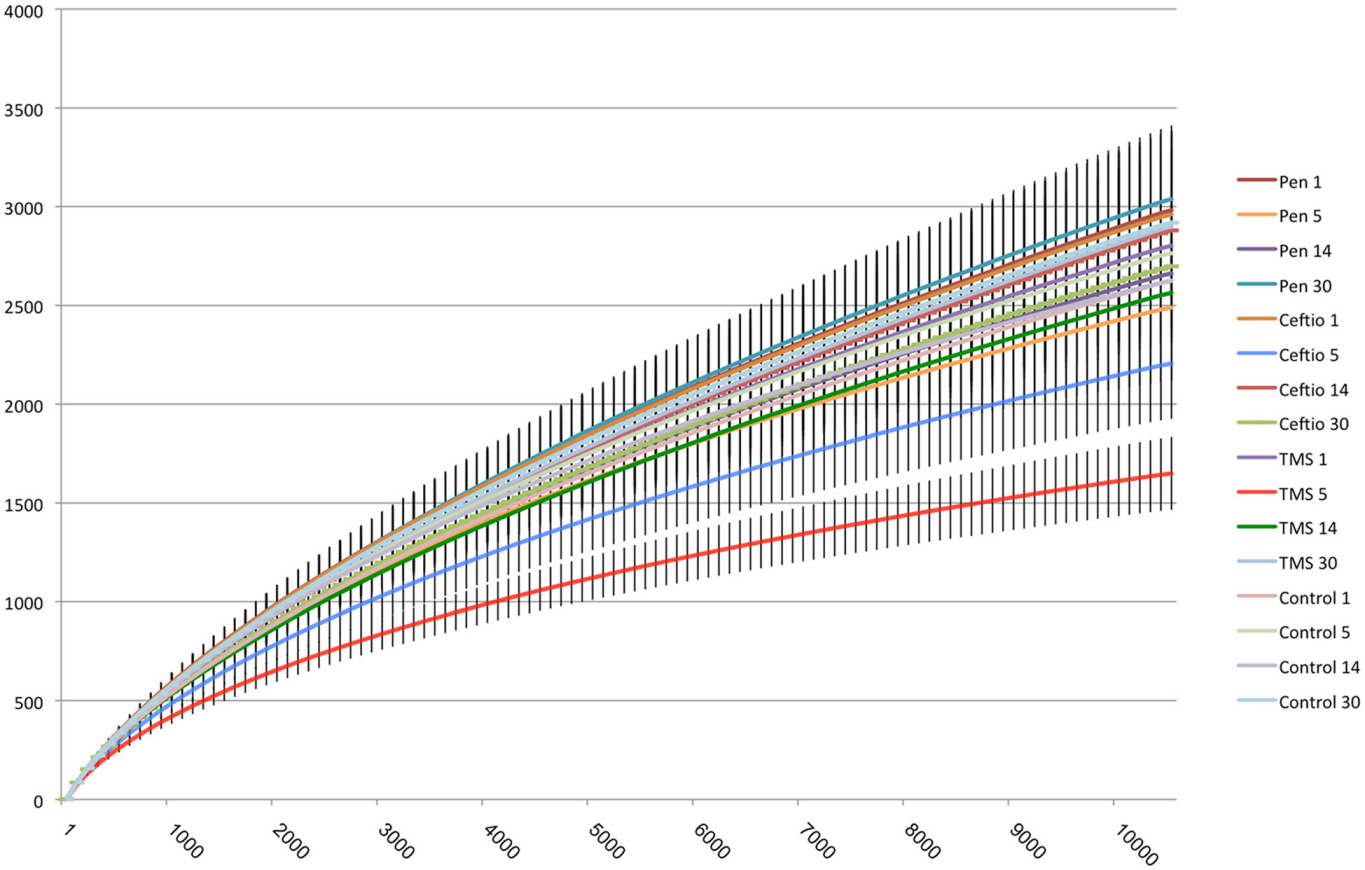
**Rarefaction curves representing the average number of OTUs (y axis) by the number of reads (x axis).**

The average of the number of reads found in each group after subsampling of 10,000 reads is represented by rarefaction curves in Figure 1. Results from the Good’s coverage achieved after subsampling are presented in Additional file 1 (mean: 85%; SD: 4%) and are also supportive of good coverage.

### Relative abundances

**Figure 2:**
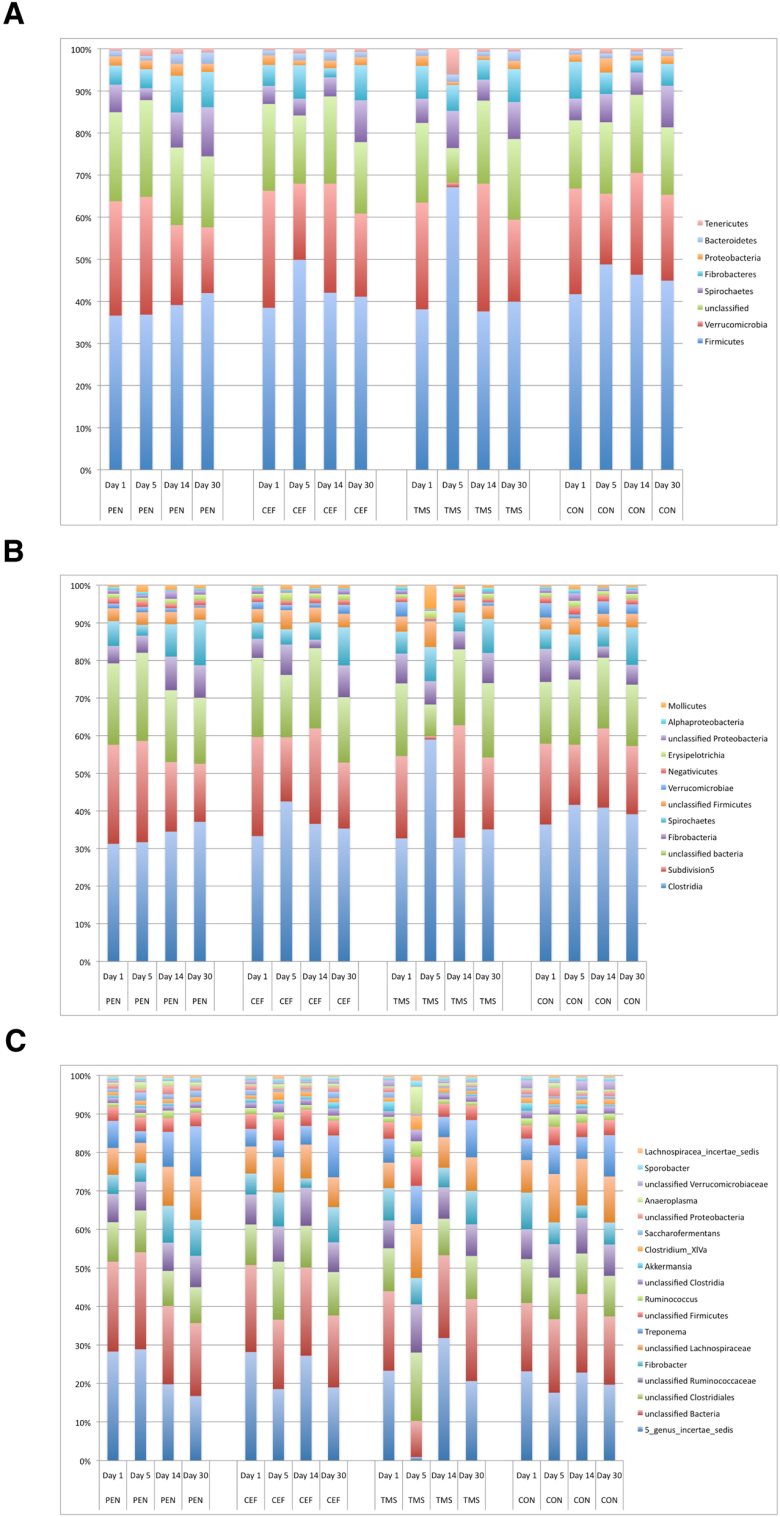
**Relative abundance of predominant bacteria at the phylum (A), class (B) and genus (C) levels.** Figure legend: penicillin (PEN), ceftiofur (CEF) and sulfa trimethoprin (TMS) and control group (CON). Day 0: before treatment; Day 5: last day of treatment.

**Figure 3:**
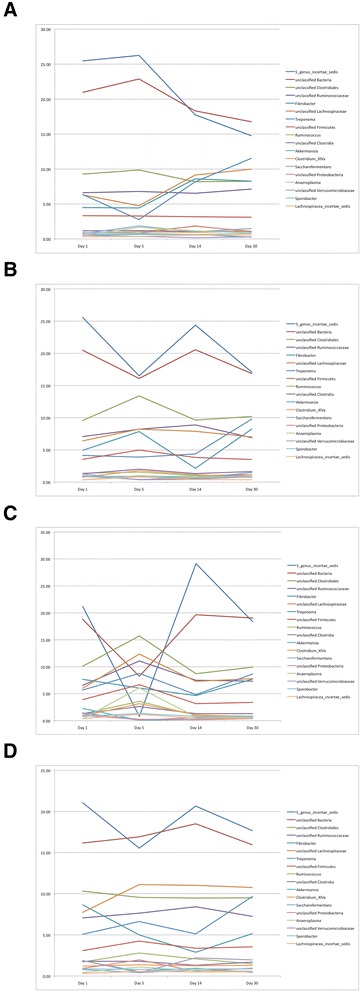
**Variation in relative abundance of predominant bacteria in feces of healthy horses treated with antibiotics.** Figure legend: **A**: penicillin; **B**: ceftiofur; **C**: sulfa trimethoprin; **D**: control group. Day 0: before treatment; Day 5: last day of treatment.

The relative abundances at the phylum, class and genus levels found in each group at the different sampling times are represented in Figure 2. Sequences were classified into 25 different phyla, of which, only eight accounted for more than 1% of sequences. The majority of bacteria found in all groups throughout the trial were assigned to the Firmicutes phylum. Verrucomicrobia represented the second main phylum, followed by bacteria that were unclassified at the phylum level. At the genus level, “5 genus *incertae sedis*”, a genus from the Subdivision 5 class of the Verrucomicrobia phylum, predominated followed by bacteria unclassified at the phylum level. Figure 3 represents variation of the main genera overtime in each treatment group.

No statistical changes in relative abundances were observed at the phylum level in response to ceftiofur administration. A decrease of Spirochetes followed by a significant increase on Day 14 (P = 0.017) was observed after treatment with penicillin. Oral TMS significantly reduced the relative abundance of Verrucomicrobia (P = 0.012), unclassified bacteria (P = 0.025) and a trend to reduce Proteobacteria (P = 0.052) and increased the abundance of Firmicutes (P = 0.012) after 5 days of treatment.

### Population analysis

Results of Simpson’s index estimating samples’ diversity, and of Catchall estimation of richness are presented in Additional file 1. Table 1 contains results from the comparison of those results at different sampling times within each group. There was a significant decrease in richness (P = 0.017) and diversity (P = 0.018) after the use of TMS (Day 1 × Day 5), but after 30 days, both estimates were similar to the beginning of the trial. No other differences in diversity or richness were identified.

**Table 1:** **P values from the Parsimony, AMOVA and t tests comparing groups at different sampling times**

**Treatment**	**Day 1- 5**	**Day 5- 14**	**Day 14- 30**	**Day 1- 14**	**Day 1- 30**
**Yue and Clayton**					
Overall					
Parsimony	<0.001	<0.001	0.137	0.005	0.053
AMOVA	<0.001	<0.001	<0.001	0.060	<0.001
Penicillin					
Parsimony	0.263	0.052	0.865	0.059	0.114
AMOVA	0.042	0.010	0.272	0.141	0.021
Ceftiofur					
Parsimony	0.004	0.065	0.704	0.305	0.298
AMOVA	0.786	0.038	0.024	0.786	0.141
TMS					
Parsimony	0.002	<0.001	0.214	0.858	0.674
AMOVA	<0.001	0.002	0.028	0.119	0.066
Control					
Parsimony	1	1	1	1	1
AMOVA	1	1	1	1	1
**Jaccard**					
Overall					
Parsimony	<0.001	0.003	0.753	0.068	0.054
AMOVA	<0.001	<0.001	0.007	<0.001	<0.001
Penicillin					
Parsimony	0.063	0.714	0.836	0.698	0.408
AMOVA	<0.001	0.012	0.669	0.172	0.260
Ceftiofur					
Parsimony	<0.001	<0.001	0.701	0.281	0.683
AMOVA	<0.001	0.006	0.140	0.037	0.074
TMS					
Parsimony	<0.001	<0.001	0.682	0.878	0.67
AMOVA	0.003	0.003	0.033	0.025	0.096
Control					
Parsimony	1	1	1	1	1
AMOVA	1	1	1	1	1
**t test**					
Penicillin					
Simpson’s	0.181	0.898	0.607		
CatchAll	0.163	0.172	0.794		
Ceftiofur					
Simpson’s	0.628	0.370	0.321		
CatchAll	0.385	0.068	0.206		
TMS					
Simpson’s	0.018	0.008	0.876		
CatchAll	0.017	0.002	0.508		
Control					
Simpson’s	0.400	0.554	0.495		
CatchAll	0.278	0.680	0.266		

**Figure 4:**
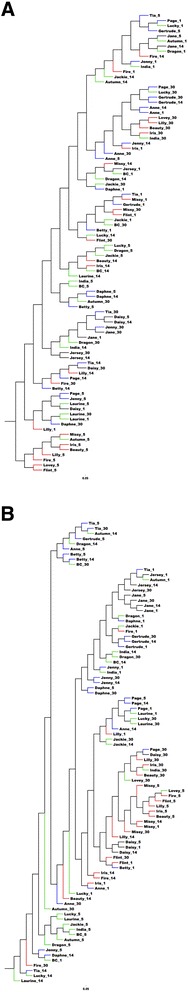
**Dendrograms representing the similarities between population structure addressed by the Yue and Clayton analysis (A) and community membership addressed by the Classic Jaccard (B).** Figure legend: Dendrograms were generated based on phylip-formatted distance matrixes using the UPGMA algorithm. The number after the name of the horse represents the day of sampling and the color of the tree brunch represents the drug used: Penicillin = blue, Ceftiofur = green, TMS = red and Control = black.

Figures 4A and B represent the dendrograms obtained with the Yue and Clayton and the Classic Jaccard analyses that respectively represent population structure (taking into account the number of OTUs and their relative abundances) and community membership (taking into account the number of OTUs). Figure 4A indicates that samples collected before treatment had more similar microbial population structure to each other and to samples collected on Day 14 and 30. In general, samples collected after treatment (Day 5) are observed at the lower part of the tree and interestingly, samples tended to cluster by the drug administered, indicating a somewhat consistent effect of each antibiotic, with the exception of animals treated with penicillin. Conversely, penicillin and ceftiofur seemed to have a strong effect on community membership, as represented by Figure 4B, in which samples from animals receiving those drugs were more distinct from other samples, even at Day 14. The changes caused by TMS and ceftiofur administration on community membership were also consistent, as samples after drug administration (Day 5) tended to cluster together.

Results from the Parsimony and AMOVA tests comparing each group at the different sampling times for either population structure and community membership are presented in Table 1. Overall, population structure and community membership were significantly different after the use of antimicrobials, regardless of the statistical test applied. The results also indicate that after 14 and 30 days population structures were still different from the beginning of the trial. Considering each group individually, penicillin had no impact on population structure and community membership evaluated by the Parsimony test, but a significant difference was identified using AMOVA. Ceftiofur and TMS induced significant changes 5 days after drug administration, but changes tended not to last for more than 14 days, indicating a recovery of population structure and community membership in the studied animals.

**Figure 5:**
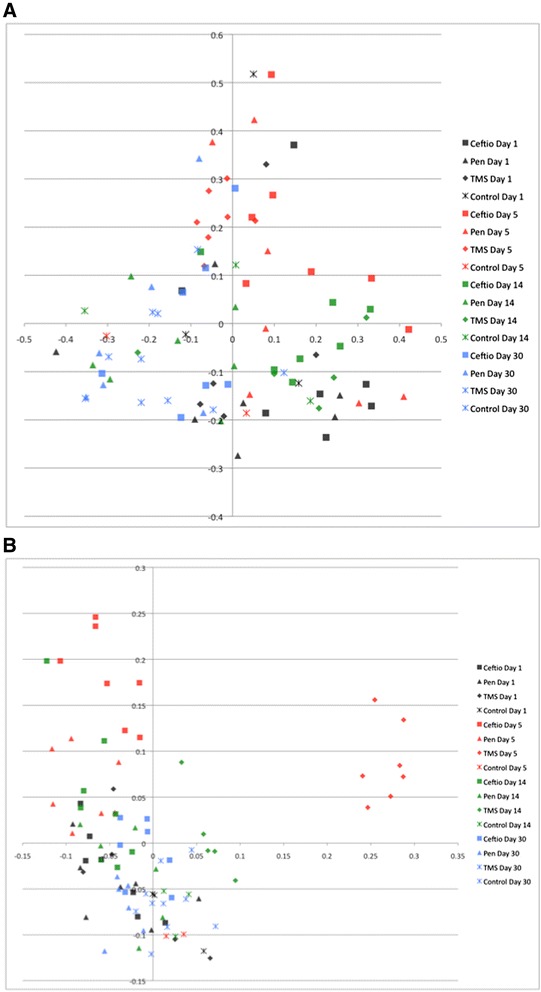
**Principal coordinate analysis (PCoA) of bacterial communities present in feces of horses treated with antibiotics.** Figure legend: Bidimentional representation of the principal coordinate analysis of bacterial communities structure addressed by the Yue and Clayton analysis **(A)** and bacterial membership addressed by the Classic Jaccard analysis **(B)** found in feces of healthy horses before (Day 1), after (Day 5) treatment with antibiotics and at Days 14 and 30.

The graphical representation of the PCoA is shown in Figure 5A and B, for the Yue and Clayton and the Classic Jaccard respectively. Despite the two first axes of the PCoA explained only 28% and 3.7% of the dissimilarities between samples respectively for the Yue and Clayton and the Classic Jaccard, clustering of samples by date of sampling and by drug administered is still evident, reinforcing the strong impact caused by antibiotics administration on the intestinal microbiota of those animals, which is further supported by the significant results from the AMOVA test (Table 1).

### Potential confounding factors

A moderate lameness was noticed in one mare in the penicillin group on Day 4 of the trial due to a sole abscess on the right front limb. The mare was transported (approximately 10 km) to the Ontario Veterinary College Health Sciences Centre (OVCHSC) after collection of the Day 5 sample and the last antimicrobial treatment was given at the hospital. The diet of that horse was unchanged (with the exception of feeding from a different batch of hay). Treatment with 2 g of phenylbutazone was given and the mare returned to the research station on Day 9. Another dose of phenylbutazone was given on Day 15, as the mare became mildly lame again. On Day 22 of the trial, another mare from the ceftiofur group was found with a deep laceration on her chest and was therefore shipped to the OVCHSC for treatment. Cleaning with topical antiseptic solution was started with no systemic antimicrobials required. The mare recovered well and was discharged from the hospital after the end of the trial.

## Discussion

Antimicrobial administration produced variable but detectable changes in the fecal microbiota. Differences were noted in specific taxonomic comparisons as well as broader evaluation of community membership (organisms present) and population structure (that takes into account the organisms present and their relative abundances). The presence of an impact of antimicrobial administration of a bacterial population is not unexpected, but the types of changes, the differences between different antimicrobials and the duration of impact are noteworthy and provide important insight.

The composition of the microbiota before antibiotic administration was not surprising, as the predominance of Firmicutes is in agreement with other studies [5,6,8,9]. The “5 genus *incertae sedis*” was the most abundant genus. This unculturable organism has been recently classified and it has been found in high abundances in feces of healthy adult horses [8,9], but its role on the equine GI tract remains to be elucidated.

The most profound effects of antimicrobials on the intestinal microbiota were observed immediately after treatment (Day 5), which is in agreement with reports in humans [20,27] and horses [15]. It was not surprising to see the main effect during antimicrobial treatment. Cessation of antimicrobial administration did not result in an immediate return to the baseline microbiota, as significant changes were still present 9 days after the end of treatment (Day 14). A similar effect has been previously observed after the use of ampicillin in humans [22] and of TMS or ceftiofur in horses [15]. By day 30 the microbiota was more similar to baseline than it was in the day 5 or day 14 samples, yet a discernable difference was still present, also evidenced by the significantly different AMOVA comparison in the group treated with penicillin (P = 0.021) and a trend in the group treated with TMS (P = 0.066). Immediate restoration of the microbiota was not expected, based on human data [21,22,27]. There was also limited apparent clustering of the day 1 and day 30 samples in the treatment group based on the dendrograms, as opposed to the control group, but further statistical comparison were not possible due to the low number of animals in the control group.It is assumed that there is more inter- than intra-individual variation and serial samples from the same individual typically cluster together [13,28], and clustering of control horse samples was evident. Here, the lack of clustering of day 1 and 30 samples from the same horse provides further evidence of an ongoing impact of antimicrobials. This provides more evidence of an ongoing impact on the microbiota, as intra-individual similarity would be expected in serial samples if the microbiota had reverted to its baseline.

Among the antimicrobials chosen for this study, TMS induced the most marked changes in population structure. Possible reasons for this are the route of administration used for this drug (oral) and its broader spectrum of action, especially when compared to penicillin, which has a narrower spectrum and is mainly excreted by the urinary tract. Oral administration can result in delivery of a large amount of active drug to the intestinal tract; however, the degree of absorption and local inactivation would have a major impact on exposure to the microbiota of the distal hindgut. Conversely, parenterally administered drugs can potentially achieve high intestinal concentrations, particularly those that undergo extensive hepatic excretion. Indeed, several classes of antimicrobials have been shown to induce changes on the luminal bacteria after intra-muscular administration [29,30] and some of the horses receiving penicillin and ceftiofur in this study had marked changes observed on community membership (Figure 4B). It is worth mentioning that the dose of ceftiofur sodium used in this study is an extra-label dose, but it was used as it reflects common dosing in the field. Further studies comparing oral versus parenteral administration of TMS would help answer the question of whether changes observed here were induced by route of administration or by the spectrum of action of this drug.

Factors such as the antimicrobial spectrum, drug levels in the gut and inactivation of the antimicrobial in the gut could all influence the impact of individual antimicrobials. TMS is one of the few drugs that horses tolerate after oral administration, and it is also available as a parenteral formulation, so comparison of the parenteral and oral routes would be useful in a future study to determine the impact of route of administration of this drug.

While TMS produced the most identifiable impacts, some degree of change was noted with all of the tested antimicrobials. A lack of understanding of the pathophysiology of antimicrobial-associated colitis and the clinical relevance of the gut microbiota hamper direct clinical assessment of the relevance of these changes. It is reasonable to postulate that more profound microbiota changes result in a greater risk of disease, yet ‘change’ and ‘clinically relevant change’ are not necessarily the same thing, and it is certainly possible that some less evident changes could be more relevant clinically. This highlights the need for more study of the intestinal microbiota in health and disease, to identify specific populations or population changes that have a greater influence.

The methods used in the present study allowed for differentiation between population structure addressed by the Yue and Clayton measure of dissimilarity that takes into account relative abundance in each sample and community membership addressed by the classic Jaccard index that takes into account the number of species. Interestingly, as it can be observed on the dendrograms, there was greater alteration of population structure compared to community membership. Thus, changes that were encountered were less likely to be addition or loss of specific community members, but rather changes in the relative abundance of existing members. This is consistent with the concept of ‘overgrowth’ of certain members in response to antimicrobial exposure.

Significant changes in the relative abundances at the phylum level observed in horses treated with penicillin and especially TMS, emphasize the potential those drugs have in causing disruption of the normal resident intestinal microbiota. Interestingly, the dramatic decrease (from 21.2% to 0.8%) of organisms classified as “5 genus *incertae sedis*” and unclassified Verrucomicrobia after the used of TMS is suggestive that this drug has a strong action against Verrucomicrobia, which allowed several genera belonging to Firmicutes to increase in abundance and it may be related to the resilience of the intestinal microbiota during recovery from severe disturbances [31]. The degree of change noted here is in contrast with earlier culture-dependent or DGGE studies, something that is not surprising because of the much greater depth that high throughput sequencing technologies allow. For instance, Gustafsson et al. [25] reported minimal effect of TMS on streptococci, Bacteroides and Veillonella counts in feces of horses, with a concurrent decrease in total coliforms. White and Prior [24] found no impact of this drug in coliforms, but a large increase of coliforms, Bacteroides, *Clostridium perfringens*, and streptococci after treatment with oxytetracycline. Moreover, Grønvold et al. [26] found no significant differences in the DGGE profile of horses after treatment with penicillin, but differences in specific bacterial groups were present in the same study when investigated by qPCR. Therefore, the conflicting results found in the literature may reflect differences based on drugs used, horse characteristics or geography, but are more likely a result of variable fidelity of the chosen methods.

Conflicting results can be found in the literature regarding the predictability of changes induced by specific antimicrobial drugs. Vancomycin has been reported to induce consistent changes on intestinal microbial structures of humans treated with that drug [32], but another study reported unpredictable changes caused by the same drug [33]. Another report [27] found different individual responses to antimicrobial therapy despite identical dosing. This disagreement might be related to the presence of other factors impacting the intestinal microbiota in those subjects. Further, since there tends to be some variability in the microbiota between individuals, it is possible that some individual microbiotas are more or less susceptible to alteration by individual antimicrobials. This study used horses that were co-housed, fed an identical diet and with other management similarities (e.g. exercise, environmental exposures), something that might minimize the inter-individual variability.

A few limitations must be considered. While consistent with many other microbiota studies, the sample size was low, which may have affected the ability to identify certain differences through limitations in statistical power. However, numerous changes were still identified. Also, we used a carefully controlled population in order to minimize other effects on the gut microbiota of these horses; therefore, caution is required for the extrapolation of our results to horses managed differently or living in different regions. In addition, it would have been interesting to continue to sample the horses in our cohort for a longer period to see when, or if, the microbiota would return to ‘normal’ for the individual animals. Finally, despite the fact that fecal microbiota have been suggested to represent the microbiota present in the large colon of horses [6], use of fecal samples may limit the study of changes occurring in more proximal compartments of the GI tract.

## Conclusions

The use of systemic antimicrobials leads to changes in the intestinal microbiota, with different and specific responses to different antimicrobials. All antimicrobials tested here had some impact on the microbiota, but TMS significantly reduced bacterial richness and diversity and had the greatest apparent impact on bacterial structures, specifically targeting members of the Verrucomicrobia phylum.

## Methods

### Animal selection

Twenty-one healthy adult horses and three ponies with no history of gastrointestinal diseases or antimicrobial administration during the previous six months were enrolled. The animals were kept on pasture and fed grass hay twice a day. All horses received hay from the same batch throughout the trial and were moved into a pen 5 days before the beginning of the trial for acclimatization. Seven horses were randomly assigned to each of three treatment groups that received procaine penicillin (20.000 UI/kg intramuscularly (IM), q12h (Pen Aqueous®, Wyeth, ON, Canada)), ceftiofur sodium (2.2 mg/kg IM, q12h (Excenel® Pfizer Animal Health, QC, Canada)) or trimethoprim sulfadiazine (TMS) (30 mg/kg orally, q12h (Uniprim Powder®, Macleod Pharmaceuticals Inc., CO, USA)) for 5 days. The sites of intra muscular injections were alternated with a maximum volume of 20 mL per site. Trimethoprim sulfadiazine was mixed with approximately 20 mL of warm corn syrup in order facilitate administration. Three individuals (one pony, one Belgian and one Thoroughbred) were assigned to the control group, which received no antimicrobials. The date of sampling, breed, age and treatment given for each horse used for the trial are presented in Table 2.

**Table 2:** **Breed, age, treatment group, and date of sampling of each studied horse**

	**Breed**	**Year of birth**	**Treatment**	**Date**
Anne	Standardbred	2006	Pen	Nov 12 – Dec 12
Betty	Standardbred	2003	Pen	Oct 9 -Nov 8
Daphne	Standardbred	2000	Pen	Sep 4 – Oct 4
Gertrude	Standardbred	2006	Pen	Sep 4 – Oct 4
Jenny	Standardbred	2000	Pen	Sep 4 – Oct 4
Paige	Standardbred	2003	Pen	Nov 12 – Dec 12
Tia	Pony	2006	Pen	Sep 4 – Oct 4
Autumn	Pony	2006	Ceftio	Sep 4 – Oct 4
Butter Cup	Standardbred	2003	Ceftio	Sep 4 – Oct 4
Dragon	Standardbred	2003	Ceftio	Sep 4 – Oct 4
India	Thoroughbred	1996	Ceftio	Sep 4 – Oct 4
Jackie	Standardbred	2007	Ceftio	Oct 9 -Nov 8
Lauryn	Standardbred	2004	Ceftio	Nov 12 – Dec 12
Lucky	Standardbred	2001	Ceftio	Nov 12 – Dec 12
Beauty	Standardbred	2004	TMS	Nov 12 – Dec 12
Fire	Standardbred	unknown	TMS	Oct 9 -Nov 8
Finch	Standardbred	1999	TMS	Oct 9 -Nov 8
Iris	Standardbred	2004	TMS	Nov 12 – Dec 12
Lilly	Belgian/Paint	2005	TMS	Nov 12 – Dec 12
Lovie	Standardbred	1997	TMS	Oct 9 -Nov 8
Missy	Standardbred	unknown	TMS	Oct 9 -Nov 8
Daisy	Belgian/Paint	2005	Control	Nov 12 – Dec 12
Jane	Standardbred	2002	Control	Sep 4 – Oct 4
Jersey	Pony	2005	Control	Sep 4 – Oct 4

Fecal samples were collected by rectal palpation using one rectal sleeve per animal. Samples were stored in plastic sterile containers and frozen at −80°C within 2 hours after collection until DNA extraction. Samples were collected before drug administration (Day 1), on the third and fifth day of treatment and again on Days 7, 14, 23 and 30 after the onset of treatment.

Table 2 lists the breed, group of treatment and dates when the trial was performed in each group. The study was approved by the University of Guelph Animal Care Committee.

### DNA extraction, 16S rRNA gene PCR and sequencing

DNA extraction was performed using a commercial kit (E.Z.N.A. Stool DNA Kit, Omega Bio-Tek Inc., USA) following the manufacturer’s “stool DNA protocol for pathogen detection”.

PCR amplification of the V4 region of the 16S rRNA gene was designed based on Klindworth et al. [34] study using the primers forward S-D-Bact-0564-a-S-15 (5′-AYTGGGYDTAAAGNG-3′) and reverse S-D-Bact-0785-b-A-18 (5′-TACNVGGGTATCTAATCC-3′). The forward and reverse primers were designed containing an overlapping region of the forward and reverse Illumina sequencing primers (TCGTCGGCAGCGTCAGATGTGTATAAGAGACAG and GTCTCGTGGGCTCGGAGATGTGTATAAGAGACAG, respectively) in order to anneal them to primers containing the Illumina adaptors plus the 8 bp identifiers indices (AATGATACGGCGACCACCGAGATCTACAC-index-TCGTCGGCAGCGTC forward and CAAGCAGAAGACGGCATACGAGAT-index-GTCTCGTGGGCTCGG reverse). For a final volume of 50 μL, 2 μL of each DNA sample was added to a solution containing 18.7 μL of water, 25 μL of Fast HotStart ReadyMix 2X (KapaBiosystems, USA), 1.3 μL of BSA (Invitrogen, USA), and 0.5 μL of each 16S primer and 1 μL of each Illumina primers (100 pmol/μL). The mixture was subjected to the following PCR conditions: 5 min at 94°C for denaturing, and 25 cycles of 30 sec at 94°C for denaturing, 30 sec at 46°C for annealing and 30 sec at 72°C for elongation followed by a final period of 7 min at 72°C and kept at 4°C until purification.

PCR products were evaluated by electrophoresis in 2% agarose gel and purified with the Agencourt AMPure XP (Beckman Coulter Inc, Mississauga, ON) by mixing 22 μL of amplicon with 72 μL of AMPure on a 96 well plate. After 5 min at room temperature, beads were separated and washed twice with 80% ethanol and eluted in 30 μL of water. After purification samples were quantified by spectrophotometry using the NanoDrop® (Roche, USA) and normalized to a final concentration of 2 nM. The library pool was sequenced with an Illumina MiSeq for 250 cycles from each end at the University of Guelph Genomics Facility.

Data was made publicly available at the NCBI Sequence Read Archive under the accession number PRJNA264726.

### Sequence analysis and statistical analysis

Bioinformatic analysis was performed using the Mothur (version 1.31.2) package of algorithms [35] following the MiSeq SOP accessed in January 2014 [36]. Briefly, original fastq files were assembled into contigs and sequences that were longer than 275 bp in length, contained any ambiguous base pairs or had runs of homopolymers greater than 8 bp were removed. Sequences were aligned using the SILVA 16S rRNA reference database [37]. Chimeras were identified and removed using uchime [38]. Sequences were then assigned into operational taxonomic units (OTUs) using a cutoff of 0.03 for the distance matrix and into phylotypes by clustering all sequences belonging to the same genus. Taxonomic classification was obtained from the Ribosomal Database Project (RDP – March 2012) [39].

A subsample from the main dataset was used for richness and diversity calculation in an attempt to decrease bias caused by non-uniform sequence depth and some low sequence number samples. The minimum number of reads that would not compromise coverage and would eliminate the fewest samples as possible from the analysis was used (10.482 reads per sample). Good’s coverage after sub-sampling was calculated in order to ensure representative sub-samples. Diversity was estimated by the inverse Simpson diversity index and richness by using CatchAll [40]. Comparison among groups was performed using a t-test. Sampling effort was evaluated by calculation of Good’s coverage and visual assessment by rarefaction curves.

The dissimilarity between groups was measured by a phylip-formated distance matrix using the Yue & Clayton measure of dissimilarity (taking into account the relative abundance of OTUs present in each group: population structure) and the classical Jaccard index (taking into account the number of shared OTUs between the groups: community membership). Dendrograms comparing the similarity of the bacterial profiles among all samples were generated using the Jaccard index and Yue & Clayton measures and figures were generated using FigTree (version 1.4.0). Population membership and structure present in the dendrograms were compared by the parsimony test.

Clustering of samples was evaluated by plotting the resultant vector of the Principal Coordinate Analysis (PCoA) with 2 dimensions. Analysis of molecular variance (AMOVA) was used to determine significance of clustering between the groups.

Bar charts representing the relative abundance at the phylum, class and genus levels of each group at the different sampling times were generated for visualization of population structure and relative abundances were compared at the different sampling times by the Steel-Dwass test controlling for multiple comparison error.

## Additional file

Additional file 1: Table S1. Good’s coverage, and alpha diversity indices after subsampling of 10.482 reads per sample.

## References

[1] Blaser MJ, Falkow S (2009). What are the consequences of the disappearing human microbiota?. Nat Publishing Group.

[2] Glinsky MJ, Smith RM, Spires HR, Davis CL (1976). Measurement of volatile fatty acid production rates in the cecum of the pony. J Anim Sci.

[3] Jassim Al RAM, Andrews FM (2009). The bacterial community of the horse gastrointestinal tract and its relation to fermentative acidosis, laminitis, colic, and stomach ulcers. Vet Clin North Am Equine Pract.

[4] Bordin AI, Suchodolski JS, Markel ME, Weaver KB, Steiner JM, Dowd SE (2013). Effects of administration of live or inactivated virulent *Rhodococccus equi* and Age on the fecal microbiome of neonatal foals. PLoS One.

[5] Costa MC, Arroyo LG, Allen-Vercoe E, Stampfli HR, Kim PT, Sturgeon A (2012). Comparison of the fecal microbiota of healthy horses and horses with colitis by high throughput sequencing of the V3-V5 region of the 16S rRNA gene. PLoS One.

[6] Dougal K, la Fuente de G, Harris PA, Girdwood SE, Pinloche E, Newbold CJ. Identification of a core bacterial community within the large intestine of the horse. PLoS ONE. 2013; 8:e77660. 10.1371/journal.pone.0077660 PMC3812009 24204908

[7] O’ Donnell MM, Harris HMB, Jeffery IB, Claesson MJ, Younge B, O’ Toole PW, Ross RP. The core faecal bacterial microbiome of Irish Thoroughbred racehorses. Lett Appl Microbiol. 2013; 57:492–501. 10.1111/lam.12137 23889584

[8] Shepherd ML, Swecker WSJ, Jensen RV, Ponder MA (2012). Characterization of the fecal bacteria communities of forage-fed horses by pyrosequencing of 16S rRNA V4 gene amplicons. FEMS Microbiol Lett.

[9] Steelman SM, Chowdhary BP, Dowd S, Suchodolski J, Janecka JE (2012). Pyrosequencing of 16S rRNA genes in fecal samples reveals high diversity of hindgut microflora in horses and potential links to chronic laminitis. BMC Vet Res.

[10] Willing BP, Voros A, Roos S, Jones C, Jansson A, Lindberg JE (2009). Changes in faecal bacteria associated with concentrate and forage-only diets fed to horses in training. Equine Vet J.

[11] Daly K, Proudman CJ, Duncan SH, Flint HJ, Dyer J, Shirazi-Beechey SP (2012). Alterations in microbiota and fermentation products in equine large intestine in response to dietary variation and intestinal disease. Br J Nutr.

[12] Kuhn M, Guschlbauer M, Feige K, Schluesener M, Bester K, Beyerbach M (2012). Feed restriction enhances the depressive effects of erythromycin on equine hindgut microbial metabolism in vitro. Berl Munch Tierarztl Wochenschr.

[13] Perkins GA, den Bakker HC, Burton AJ, Erb HN, McDonough SP, McDonough PL (2012). Equine stomachs harbor an abundant and diverse mucosal microbiota. Appl Environ Microbiol.

[14] Faubladier C, Chaucheyras-Durand F, da Veiga L, Julliand V (2013). Effect of transportation on fecal bacterial communities and fermentative activities in horses: Impact of *Saccharomyces cerevisiae* CNCM I-1077 supplementation. J Anim Sci.

[15] Harlow BE, Lawrence LM, Flythe MD (2013). Diarrhea-associated pathogens, lactobacilli and cellulolytic bacteria in equine feces: Responses to antibiotic challenge. Vet Microbiol.

[16] Chapman AM (2009). Acute diarrhea in hospitalized horses. Vet Clin North Am Equine Pract.

[17] Barr BS, Waldridge BM, Morresey PR, Reed SM, Clark C, Belgrave R (2013). Antimicrobial-associated diarrhoea in three equine referral practices. Equine Vet J.

[18] Cohen ND, Woods AM (1999). Characteristics and risk factors for failure of horses with acute diarrhea to survive: 122 cases (1990–1996). J Am Vet Med Assoc.

[19] Cochetière MF, Durand T, Lalande V, Petit JC, Potel G, Beaugerie L (2008). Effect of antibiotic therapy on human fecal microbiota and the relation to the development of *Clostridium difficile*. Microb Ecol.

[20] Dethlefsen L, Relman DA (2011). Incomplete recovery and individualized responses of the human distal gut microbiota to repeated antibiotic perturbation. Proc Natl Acad Sci U S A.

[21] Janczyk P, Pieper R, Souffrant WB, Bimczok D, Rothkötter H-J, Smidt H (2007). Parenteral long-acting amoxicillin reduces intestinal bacterial community diversity in piglets even 5 weeks after the administration. ISME J.

[22] Perez-Cobas AE, Gosalbes MJ, Friedrichs A, Knecht H, Artacho A, Eismann K (2013). Gut microbiota disturbance during antibiotic therapy: a multi-omic approach. Gut.

[23] Jakobsson HE, Jernberg C, Andersson AF, Sjölund-Karlsson M, Jansson JK, Engstrand L (2010). Short-term antibiotic treatment has differing long-term impacts on the human throat and Gut microbiome. PLoS One.

[24] White G, Prior SD (1982). Comparative effects of oral administration of trimethoprim/sulphadiazine or oxytetracycline on the faecal flora of horses. Vet Rec.

[25] Gustafsson A, Baverud V, Franklin A, Gunnarsson A, Ogren G, Ingvast-Larsson C (1999). Repeated administration of trimethoprim/sulfadiazine in the horse–pharmacokinetics, plasma protein binding and influence on the intestinal microflora. J Vet Pharmacol Ther.

[26] GrÃ nvold A-MR, L’AbÃ e-Lund TM, SÃ rum H, Skancke E, Yannarell AC, Mackie RI. Changes in fecal microbiota of healthy dogs administered amoxicillin. FEMS Microbiol Ecol. 2010; 71:313–326. 10.1111/j.1574-6941.2009.00808.x 20002181

[27] La Cochetiere De MF, Durand T, Lepage P, Bourreille A, Galmiche JP, Doré J (2005). Resilience of the dominant human fecal microbiota upon short-course antibiotic challenge. J Clin Microbiol.

[28] Blackmore TM, Dugdale A, Argo CM, Curtis G, Pinloche E, Harris PA (2013). Strong stability and host specific bacterial community in faeces of ponies. PLoS One.

[29] Ferran AA, Bibbal D, Pellet T, Laurentie M, Gicquel-Bruneau M, Sanders P (2013). Pharmacokinetic/pharmacodynamic assessment of the effects of parenteral administration of a fluoroquinolone on the intestinal microbiota: comparison of bactericidal activity at the gut versus the systemic level in a pig model. Int J Antimicrob Agents.

[30] Tanayama S, Yoshida K, Adachi K, Kondo T (1980). Metabolic fate of SCE-1365, a new broad-spectrum cephalosporin, after parenteral administration to rats and dogs. Antimicrob Agents Chemother.

[31] Peris-Bondia F, Latorre A, Artacho A, Moya A, D’Auria G (2011). The active human Gut microbiota differs from the total microbiota. PLoS One.

[32] Robinson CJ, Young VB (2010). Antibiotic administration alters the community structure of the gastrointestinal micobiota. Gut Microbes.

[33] Morotomi N, Fukuda K, Nakano M, Ichihara S, Oono T, Yamazaki T (2011). Evaluation of intestinal microbiotas of healthy Japanese adults and effect of antibiotics using the 16S ribosomal RNA gene based clone library method. Biol Pharm Bull.

[34] Klindworth A, Pruesse E, Schweer T, Peplies J, Quast C, Horn M (2013). Evaluation of general 16S ribosomal RNA gene PCR primers for classical and next-generation sequencing-based diversity studies. Nucleic Acids Res.

[35] Schloss PD, Westcott SL, Ryabin T, Hall JR, Hartmann M, Hollister EB (2009). Introducing mothur: open-source, platform-independent, community-supported software for describing and comparing microbial communities. Appl Environ Microbiol.

[36] Kozich JJ, Westcott SL, Baxter NT, Highlander SK, Schloss PD (2013). Development of a dual-index sequencing strategy and curation pipeline for analyzing amplicon sequence data on the MiSeq illumina sequencing platform. Appl Environ Microbiol.

[37] Quast C, Pruesse E, Yilmaz P, Gerken J, Schweer T, Yarza P (2013). The SILVA ribosomal RNA gene database project: improved data processing and web-based tools. Nucleic Acids Res.

[38] Edgar RC, Haas BJ, Clemente JC, Quince C, Knight R (2011). UCHIME improves sensitivity and speed of chimera detection. Bioinformatics.

[39] Cole JR, Wang Q, Fish JA, Chai B, McGarrell DM, Sun Y (2014). Ribosomal database Project: data and tools for high throughput rRNA analysis. Nucleic Acids Res.

[40] Bunge J: Estimating the number of species with CatchAll. Pac Symp Biocomput 2011:121–130. 10.1142/9789814335058_0014 21121040

