# Supplementary material for: Changes in the equine fecal microbiota associated with the use of systemic antimicrobial drugs
Source: BMC Vet Res. 2015 Feb 3;11:19. doi: 10.1186/s12917-015-0335-7 (PMC4323147; doi:10.1186/s12917-015-0335-7)
Supplement: Additional file 1: Table S1. — Good’s coverage, and alpha diversity indices after subsampling of 10.482 reads per sample. [file 12917_2015_335_MOESM1_ESM.docx]

Additional file 1: Table S1- Good’s coverage, and alpha diversity indices after subsampling of 10.482 reads per sample.

| Group | Coverage | OTUs | Catchall | Simpson |
| --- | --- | --- | --- | --- |
| Anne_1 | 0.827 | 3043 | 9657.4 | 202.3 |
| Anne_14 | 0.845 | 2581 | 12696.9 | 136.4 |
| Anne_30 | 0.82 | 3376 | 8654.2 | 393.5 |
| Anne_5 | 0.83 | 2806 | 12386.9 | 242.7 |
| Autumn_1 | 0.849 | 2844 | 7856.5 | 370.0 |
| Autumn_14 | 0.806 | 3216 | 20083.2 | 422.3 |
| Autumn_30 | 0.87 | 2546 | 6546.9 | 214.7 |
| Autumn_5 | 0.905 | 1924 | 5000.7 | 85.9 |
| BC_1 | 0.789 | 3431 | 14474.6 | 200.7 |
| BC_14 | 0.813 | 3051 | 17362.7 | 255.6 |
| BC_30 | 0.801 | 3269 | 14977.3 | 268.2 |
| BC_5 | 0.858 | 2474 | 10086.2 | 193.9 |
| Beauty_14 | 0.86 | 2343 | 13421.2 | 131.4 |
| Beauty_30 | 0.806 | 3079 | 20607.9 | 228.9 |
| Beauty_5 | 0.929 | 1722 | 3515.1 | 208.9 |
| Betty_1 | 0.877 | 2360 | 6732.9 | 175.4 |
| Betty_14 | 0.837 | 2665 | 24206.8 | 113.5 |
| Betty_5 | 0.898 | 2386 | 4787.7 | 272.2 |
| Daisy_1 | 0.858 | 2519 | 8592 | 46.0 |
| Daisy_14 | 0.885 | 2512 | 5475.5 | 222.9 |
| Daisy_30 | 0.833 | 2867 | 11314.4 | 177.0 |
| Daisy_5 | 0.838 | 2870 | 9053.7 | 327.2 |
| Daphne_1 | 0.833 | 2926 | 14210.3 | 385.5 |
| Daphne_14 | 0.892 | 2703 | 5047.4 | 315.1 |
| Daphne_30 | 0.85 | 2551 | 10776.6 | 77.1 |
| Daphne_5 | 0.857 | 2491 | 12313 | 209.3 |
| Dragon_1 | 0.845 | 2922 | 8960.7 | 323.3 |
| Dragon_14 | 0.806 | 3120 | 18085.4 | 299.9 |
| Dragon_30 | 0.832 | 2874 | 10182.1 | 267.7 |
| Dragon_5 | 0.912 | 2163 | 4099.8 | 134.4 |
| Fire_1 | 0.789 | 3456 | 16776.2 | 367.0 |
| Fire_14 | 0.853 | 2542 | 11337.8 | 216.0 |
| Fire_30 | 0.836 | 2905 | 9146.4 | 151.2 |
| Fire_5 | 0.915 | 1737 | 6983.4 | 55.7 |
| Flint_1 | 0.843 | 2843 | 8976.1 | 266.8 |
| Flint_30 | 0.871 | 2373 | 7196.5 | 158.9 |
| Flint_5 | 0.912 | 1772 | 5469.1 | 51.6 |
| Gertrude_1 | 0.773 | 3628 | 22863.9 | 452.2 |
| Gertrude_14 | 0.812 | 3056 | 15501.2 | 194.8 |
| Gertrude_30 | 0.788 | 3300 | 18529.6 | 224.7 |
| Gertrude_5 | 0.83 | 2841 | 13836.4 | 184.4 |
| India_1 | 0.837 | 2821 | 11488.8 | 217.3 |
| India_14 | 0.818 | 2986 | 29653.1 | 147.9 |
| India_30 | 0.832 | 2851 | 14754.2 | 212.2 |
| India_5 | 0.844 | 2586 | 19931.4 | 222.7 |
| Iris_1 | 0.861 | 2697 | 7136.5 | 226.0 |
| Iris_14 | 0.84 | 2805 | 9863.1 | 221.3 |
| Iris_30 | 0.789 | 3371 | 22941.1 | 281.3 |
| Iris_5 | 0.928 | 1693 | 3467.6 | 135.5 |
| Jackie_1 | 0.773 | 3617 | 17128.2 | 420.9 |
| Jackie_14 | 0.82 | 3061 | 11912.1 | 310.9 |
| Jackie_30 | 0.869 | 2358 | 9247 | 174.9 |
| Jackie_5 | 0.869 | 2392 | 8882.4 | 158.4 |
| Jane_1 | 0.845 | 2727 | 10376.9 | 304.7 |
| Jane_14 | 0.834 | 2860 | 11685.1 | 311.6 |
| Jane_30 | 0.819 | 3025 | 15433.8 | 265.0 |
| Jane_5 | 0.859 | 2657 | 8312.2 | 227.1 |
| Jenny_1 | 0.825 | 2995 | 12540.3 | 309.9 |
| Jenny_14 | 0.867 | 2383 | 7880.9 | 148.3 |
| Jenny_30 | 0.823 | 2991 | 15858.4 | 174.4 |
| Jenny_5 | 0.937 | 1926 | 3112.5 | 140.1 |
| Jersey_1 | 0.851 | 2631 | 11316.2 | 183.0 |
| Jersey_14 | 0.893 | 2502 | 5112.3 | 163.4 |
| Jersey_30 | 0.85 | 2863 | 7585.4 | 120.7 |
| Laurine_1 | 0.841 | 2678 | 10524.5 | 78.7 |
| Laurine_14 | 0.919 | 1685 | 3819.5 | 68.7 |
| Laurine_30 | 0.848 | 2578 | 13541.6 | 129.2 |
| Laurine_5 | 0.893 | 1884 | 7716.3 | 27.4 |
| Lilly_1 | 0.879 | 2089 | 9388.7 | 54.6 |
| Lilly_14 | 0.849 | 2648 | 16627.1 | 211.5 |
| Lilly_30 | 0.836 | 2773 | 12955 | 121.6 |
| Lilly_5 | 0.899 | 1838 | 8938.1 | 73.7 |
| Lovey_30 | 0.855 | 2517 | 9743.5 | 151.8 |
| Lovey_5 | 0.93 | 1453 | 4333.5 | 19.5 |
| Lucky_1 | 0.874 | 2411 | 7433.3 | 136.1 |
| Lucky_14 | 0.807 | 3034 | 17288.6 | 270.1 |
| Lucky_30 | 0.855 | 2416 | 15291 | 117.7 |
| Lucky_5 | 0.889 | 2016 | 6971.7 | 82.5 |
| Missy_1 | 0.827 | 2921 | 18274.5 | 333.1 |
| Missy_14 | 0.861 | 2481 | 9480.5 | 201.8 |
| Missy_30 | 0.797 | 3275 | 16604.6 | 322.9 |
| Missy_5 | 0.943 | 1333 | 3884.1 | 93.4 |
| Page_1 | 0.823 | 2936 | 15429.6 | 175.4 |
| Page_14 | 0.848 | 2590 | 10786.7 | 204.6 |
| Page_30 | 0.82 | 2971 | 20449.4 | 202.2 |
| Page_5 | 0.849 | 2672 | 9056.9 | 158.6 |
| Tia_1 | 0.825 | 2991 | 11346.7 | 275.7 |
| Tia_14 | 0.786 | 3445 | 29972 | 332.6 |
| Tia_30 | 0.821 | 3200 | 10060.4 | 345.3 |
